# Idebenone and coenzyme Q_10_ are novel PPARα/γ ligands, with potential for treatment of fatty liver diseases

**DOI:** 10.1242/dmm.034801

**Published:** 2018-08-31

**Authors:** Jens Tiefenbach, Lilia Magomedova, Jiabao Liu, Arkadiy A. Reunov, Ricky Tsai, Neena S. Eappen, Rebecca A. Jockusch, Corey Nislow, Carolyn L. Cummins, Henry M. Krause

**Affiliations:** 1 University of Toronto, Donnelly Ctr., 160 College St, Toronto, ON M5S 3E1, Canada; 2 InDanio Bioscience Inc., 160 College Street, Toronto, ON M5S 3E1, Canada; 3 Department of Pharmaceutical Sciences, Leslie Dan Faculty of Pharmacy, 144 College St, University of Toronto, Toronto, ON M5S 3M2, Canada; 4 Department of Chemistry, 80 St George St, University of Toronto, Toronto, ON M5S 3H4, Canada; 5 The University of British Columbia, Faculty of Pharmaceutical Sciences, 2405 Wesbrook Mall, Vancouver, BC V6T 1Z3, Canada; 6 Department of Molecular Genetics, University of Toronto, Toronto, ON M5S 1A8, Canada

**Keywords:** Drug discovery, Fatty liver disease, Idebenone, Nuclear receptors, Zebrafish

## Abstract

Current peroxisome proliferator-activated receptor (PPAR)-targeted drugs, such as the PPARγ-directed diabetes drug rosiglitazone, are associated with undesirable side effects due to robust agonist activity in non-target tissues. To find new PPAR ligands with fewer toxic effects, we generated transgenic zebrafish that can be screened in high throughput for new tissue-selective PPAR partial agonists. A structural analog of coenzyme Q_10_ (idebenone) that elicits spatially restricted partial agonist activity for both PPARα and PPARγ was identified. Coenzyme Q_10_ was also found to bind and activate both PPARs in a similar fashion, suggesting an endogenous role in relaying the states of mitochondria, peroxisomes and cellular redox to the two receptors. Testing idebenone in a mouse model of type 2 diabetes revealed the ability to reverse fatty liver development. These findings indicate new mechanisms of action for both PPARα and PPARγ, and new potential treatment options for nonalcoholic fatty liver disease (NAFLD) and steatosis.

This article has an associated First Person interview with the first author of the paper.

## Abstract (precis)

**Summary:** A zebrafish screen identifies a novel PPARα/γ ligand, idebenone, with potential for treatment of fatty liver diseases, as seen by testing it in a mouse model of type 2 diabetes.

## INTRODUCTION

Obesity and associated metabolic disorders have become an epidemic worldwide with some of the most costly and deadly outcomes (Browning et al., 2004). Obese individuals have an increased risk for developing fatty liver (hepatic steatosis), abnormal lipid levels in the blood (dyslipidemia), atherosclerosis and type 2 diabetes (T2D) (Browning et al., 2004; Cohen et al., 2011; Samuel and Shulman, 2012). One of the most effective and immediate means of treating these diseases is through the use of small-molecule regulators of the PPAR (NR1C) family of nuclear receptors (NRs) (Shearer and Billin, 2007).

In humans, there are three, closely related, PPAR genes: *PPARA*, *PPARD* and *PPARG* (Wang, 2010). The encoded receptors exert major roles in the control of energy metabolism via distinct yet overlapping tissue distributions and functions (Wang et al., 2003). Ligand binding to PPARs, and heterodimerization with retinoid X receptors (RXRs), results in the recruitment of co-activators to target gene response elements and transcriptional activation (Fan and Evans, 2015; Perissi and Rosenfeld, 2005).

PPARs belong to the ‘adopted orphan’ NR subgroup, for which endogenous ligands have been identified, although for some their physiological relevance is still unclear (Wang, 2010; Chakravarthy et al., 2009). PPARα is expressed strongly in all metabolic tissues, but is highest in the liver where agonist binding promotes lipolysis and fatty acid oxidation (Braissant et al., 1996). Accordingly, fibrates, a class of amphipathic carboxylic acids that target PPARα, are widely used as lipid-lowering agents. PPARδ is involved in the regulation of fatty acid oxidation and mitochondrial biogenesis predominantly in skeletal muscle, liver and adipose tissue (Wang et al., 2003). PPARγ is considered to be the master regulator of adipogenesis, via its promotion of lipid production and storage (Tontonoz and Spiegelman, 2008). The most effective PPARγ drugs are the thiazolidinediones (TZDs) rosiglitazone and pioglitazone, which are most widely used for the treatment of T2D (Olefsky, 2000). However, the strong agonist activities of these drugs cause harmful, off-target side effects such as weight gain, edema, osteoporosis, heart failure and cancer (Abbas et al., 2012; Shah and Mudaliar, 2010). Recently, it has been shown that PPARγ ligands that have partial or no agonist activity still have the insulin-sensitizing activities of TZDs without the adverse side effects (Choi et al., 2011).

The use of zebrafish (*Danio rerio*) as an animal model to study development and disease has revealed many advantages that make it ideal for *in vivo* drug testing and screening (Peterson et al., 2000; Delvecchio et al., 2011). Previously, we developed a zebrafish screening platform that allows the screening and validation of human nuclear receptor modulators in live fish (Delvecchio et al., 2011; Tiefenbach et al., 2010). Treatment of the transparent offspring with active compounds results in expression of green fluorescent protein (GFP) in responsive tissues. Here, we made use of our hPPARγ line to carry out a high-content chemical screen. One of the hits, idebenone, developed as a soluble analog of coenzyme Q_10_ (CoQ_10_), suggested that CoQ_10_ may serve as an endogenous ligand for PPARs. Indeed, we found that CoQ_10_ and many of its derivatives serve as direct partial agonists, not only for PPARγ but also for PPARα. While idebenone is able to regulate the expression of a subset of known PPARα and PPARγ target genes, unlike TZDs, it does not induce lipogenesis. In obese mice, treatment with idebenone resulted in reversal of fatty liver development, whereas treatment with rosiglitazone exacerbated the problem.

## RESULTS

### First whole-animal *in vivo* drug screen for human PPARs

**Fig. 1.**
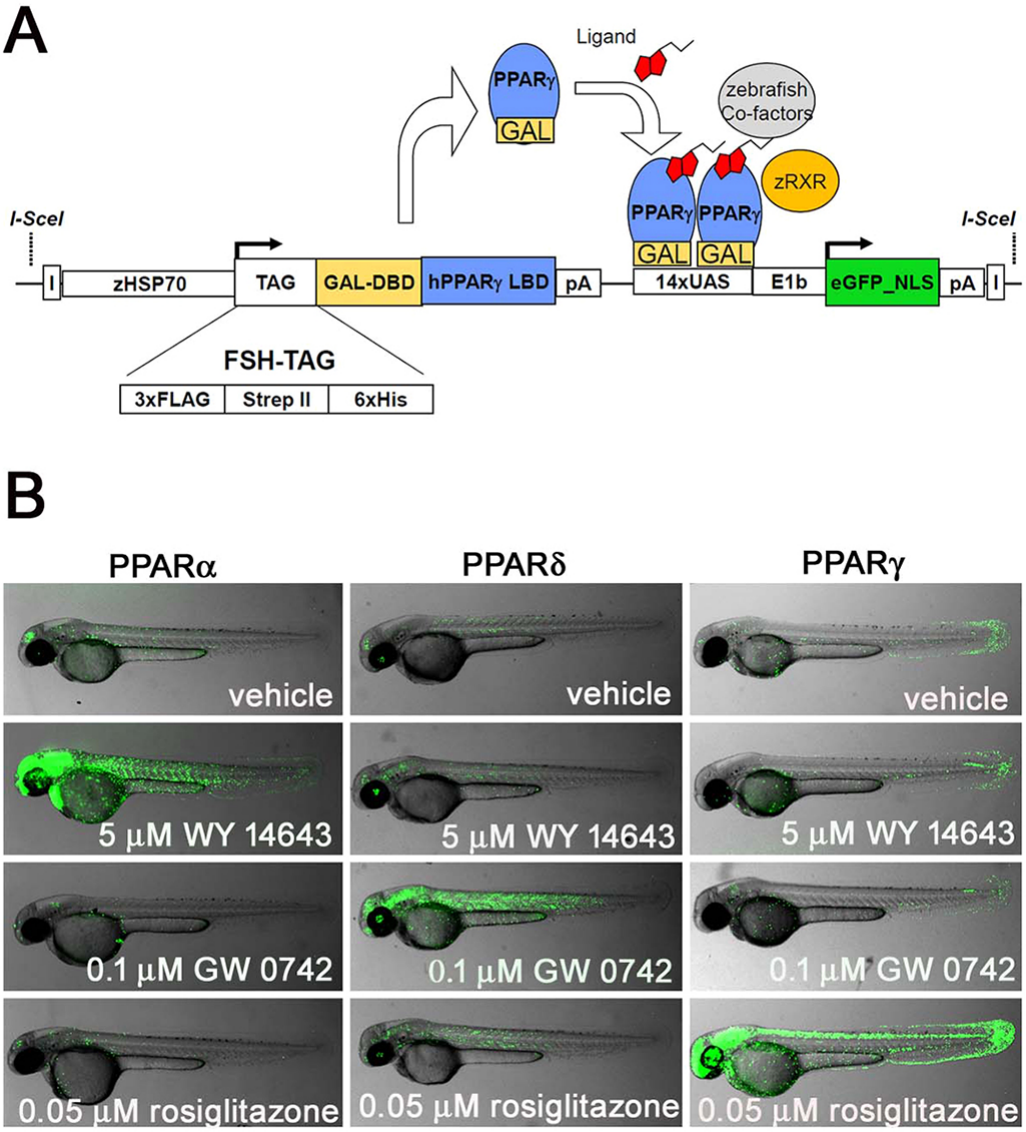
**PPAR LT fish.** (A) In the ligand trap (LT) system (Chawla et al., 1994), a DNA-binding domain (DBD)–human nuclear receptor ligand-binding domain (LBD) fusion protein is used to signal the presence of ligand *in vivo*. In the presence of specific ligands and cofactors, binding of the fusion protein to a GFP reporter results in expression of GFP. Previously published by Tiefenbach et al. (2010) under a CC-BY license (https://creativecommons.org/licenses/by/4.0/). (B) 2 dpf PPARα, PPARδ and PPARγ LT fish respond to endogenous fish hormones and PPAR-specific agonists. Active concentrations were similar to those observed in human cells, and non-specific cross-receptor activities were not observed for any of the tested agonists on the other two PPAR receptors (mean *n*=5).

We have expanded our previously described ‘ligand trap’ (LT) system for human NR studies (Tiefenbach et al., 2010) to include all three human PPAR family members. The bipartite LT system contains a heterologous DNA-binding domain (DBD) fused in-frame to a human NR ligand-binding domain (LBD). Expression of the fusion protein is under the control of the inducible zebrafish *hsp70* promoter. Upon induction, and in tissues containing active ligands and cofactor orthologs, the fusion protein is able to activate expression of an enhanced GFP (eGFP) reporter (Fig. 1A; Tiefenbach et al., 2010).

**Fig. 2.**
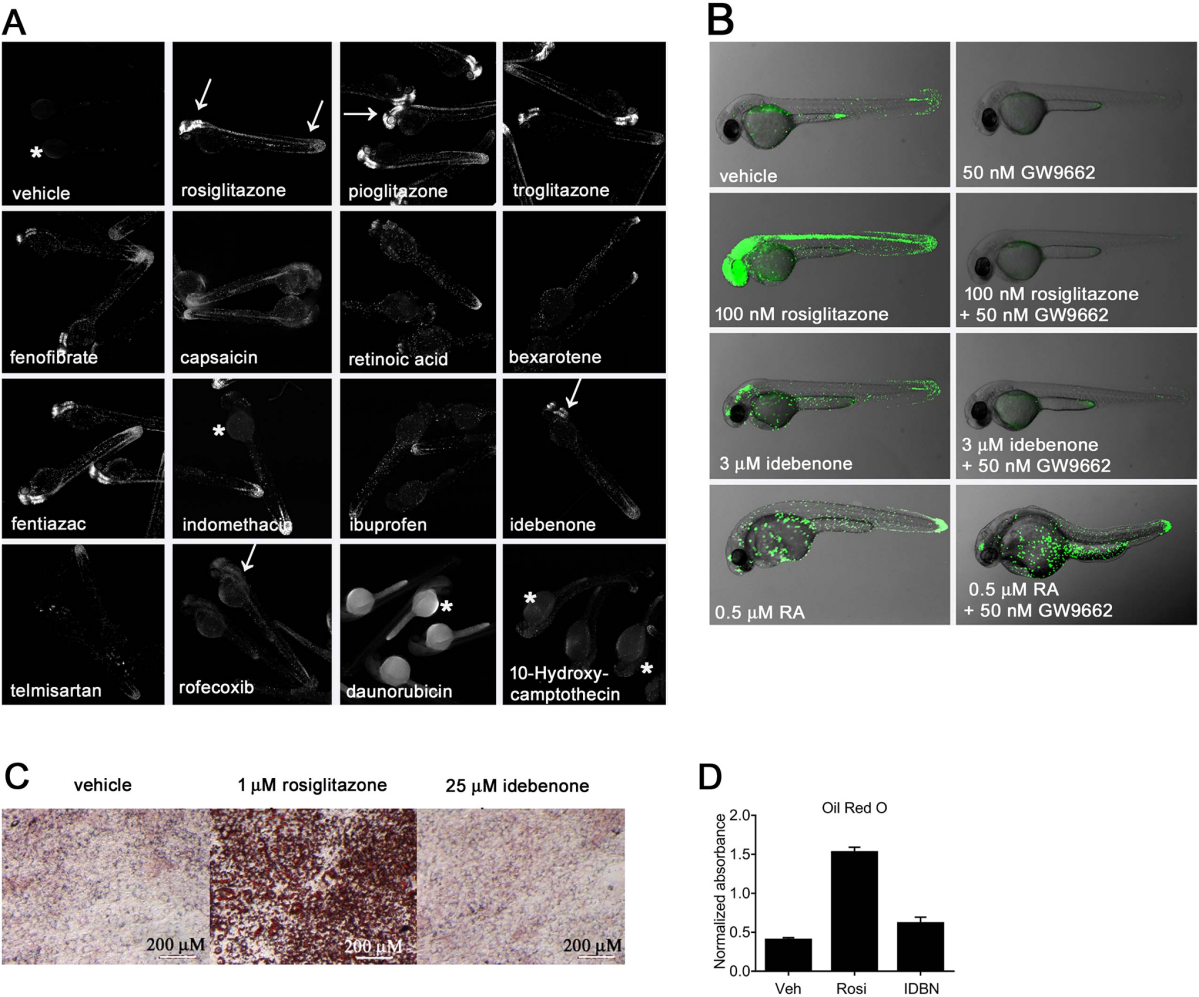
**Analysis of hits.** (A) Pictures of laser-scanning cytometer-imaged hits in 2 dpf embryos. 1% DMSO was used as vehicle and 50 nM rosiglitazone as positive control. At 1 µM screening concentration, 12 hits were identified. Images show close ups from re-screens at 10 µM concentrations. Idebenone and false positives (last two images) were treated with 3 µM concentrations due to lethality at 10 µM (mean *n*=5, replicated two times). Arrows indicate sites of GFP expression. Asterisks indicate nonspecific background. (B) At the stage shown (2 dpf), vehicle-treated LT-PPARγ transgenic embryos show weak responses to endogenous ligands and cofactors and strong responses in the presence of the receptor-specific full agonist rosiglitazone in the CNS, heart, blood, renal tube and eye. Idebenone treatment leads to relatively lower and more restricted GFP expression in cells of the blood, epidermis and CNS. 9-*cis-*retinoic acid (RA) increases GFP expression in keratinocytes, in the tail bud and in epidermis, presumably through activation of the zebrafish RXR receptor. The GW9662 suicide antagonist blocks agonist activity of rosiglitazone and idebenone, but not of RA. Overlay pictures of bright-field and GFP (85% transparent) from 2 dpf embryos are shown. Views are lateral with anterior to the left (mean *n*=10, replicated two times). (C) The treatment of murine 3T3-L1 pre-adipocytes with rosiglitazone in the presence of dexamethasone, isobutylmethylxanthine and insulin results in the accumulation of intracellular lipids. In contrast, cells treated with vehicle (DMSO) or idebenone (IDBN) instead of rosiglitazone showed no accumulation of lipid (Oil-Red-O staining). Images were taken at 100× magnification with a Leica M205 FA microscope. (D) Oil-Red-O staining quantification from cells shown in C. Oil-Red-O dye was eluted using isopropanol and absorbance was measured at 505 nm. Data represent mean±s.d., *n*=3.

The three PPAR fish lines generated here respond to endogenous zebrafish small molecules (i.e. metabolites) and cofactors in a tissue-specific manner (Fig. 1B). At 2 days post-fertilization (dpf), embryos with ubiquitous PPARα fusion-gene expression produce GFP in the forebrain, retina and epidermis (Fig. 1B and data not shown). In PPARδ LT embryos, GFP expression occurs in skeletal muscle and the retina (Fig. 1B and data not shown). With PPARγ LT embryos, GFP is produced in keratinocytes, the retina and the posterior spinal cord (Fig. 1B, Fig. S1; Chawla et al., 1994). In the presence of exogenously added PPARα- and PPARγ-specific full agonists (WY 14643 and rosiglitazone, respectively), GFP expression expands from the relatively restricted patterns seen in the absence of drugs to include strong expression in the CNS, epidermis, heart, blood and retina (Fig. 1B, Fig. S1; Tiefenbach et al., 2010). PPARα LT embryos also show strong expression in muscle. The PPARδ agonist (GW 0742) increases activity in skeletal muscle and brain (Fig. 1B and data not shown). Typical levels of signal variability are shown in Fig. S1. Note that the eGFP reporter protein, which includes a nuclear localization signal, results in dot-like expression patterns (indicated by arrows in Fig. 2A), as opposed to the diffuse auto-fluorescent signals generated by the yolk and necrotic tissues when viewed with relatively high exposures (indicated by asterisks in Fig. 2A and Fig. S1).

### Idebenone, a novel PPARγ modulator

As a first validation of the potential of our fish lines for *in vivo* drug discovery, we conducted a screen to identify PPARγ-selective partial agonists (Fig. 2A). A 640-compound library of Food and Drug Administration (FDA)-approved drugs was screened in duplicate at a concentration of 1 µM. Increases in GFP expression relative to vehicle-treated embryos suggests agonist activity for test compounds. A total of 12 hits (1.88% hit rate and 1.25% lethality rate) were obtained (Fig. 2A). Nine previously known PPARγ agonists present in the library were identified (Table S1). Among these were the well-known PPARγ TZD drugs rosiglitazone, troglitazone and pioglitazone, as well as several nonsteroidal anti-inflammatory drugs and capsaicin (N-vanillyl-8-methyl-alpha-nonenamide), a natural component of hot peppers. Fenofibrate, a PPARα selective agonist known to activate PPARγ at micromolar concentrations, was also identified (Fig. 2A). The selective retinoid X receptor (RXR) agonists bexarotene and retinoic acid also weakly activated the PPARγ reporter fish, but with a more mosaic response pattern, due presumably to activation of endogenous zebrafish RXRs recruited to the reporter by the PPARγ LBD.

**Fig. 3.**
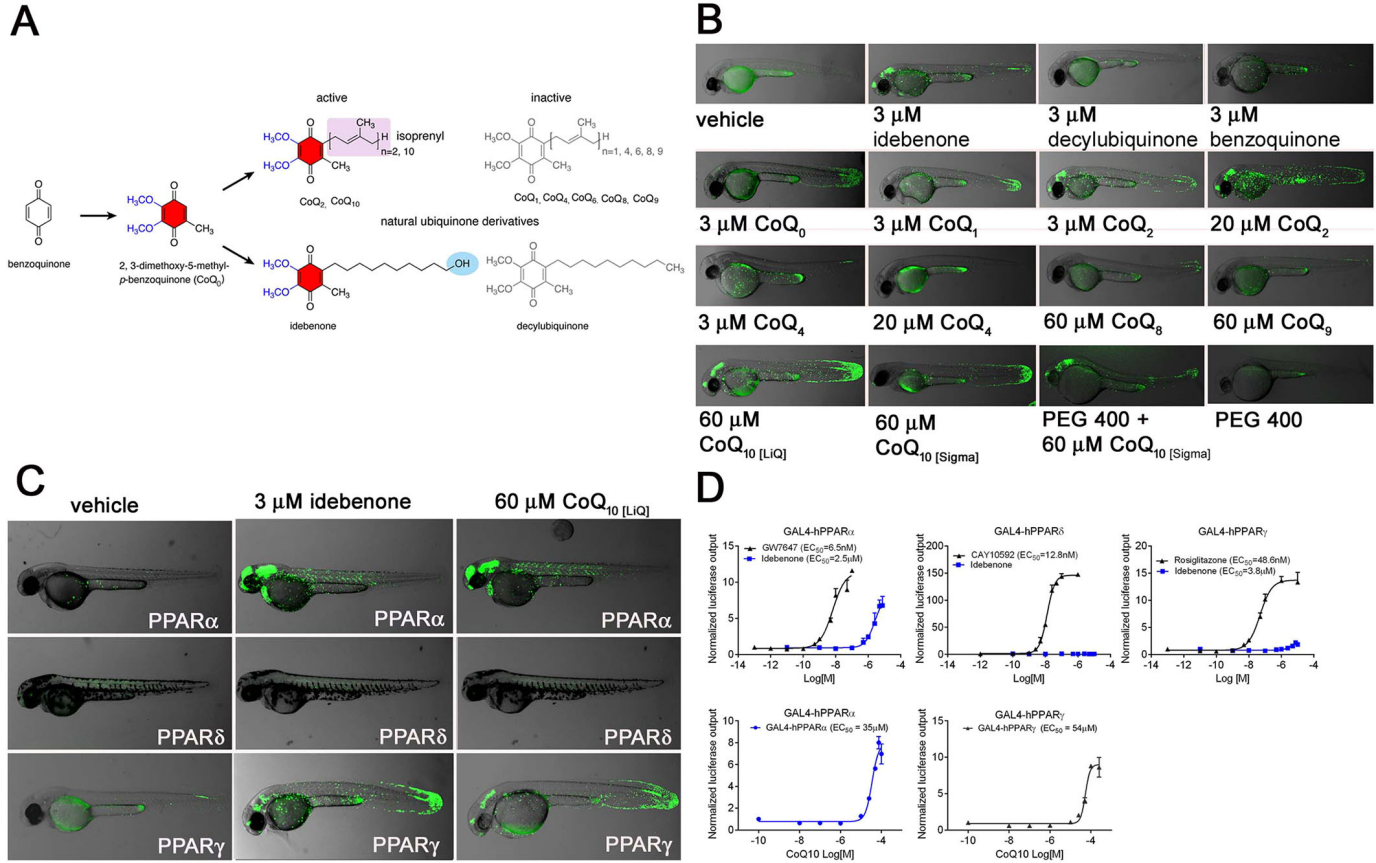
**Benzoquinone structures and hPPAR activities.** (A) Structures of idebenone, CoQ_10_ and derivatives. Note that the isoprenoid side chain contains ten isoprenoid subunits, whereas idebenone contains only alkyl units and ends with a polar hydroxyl group. (B) 2 dpf PPARγ LT embryos showing limited GFP expression in a small subset of epidermal cells and in the posterior spinal cord in the absence of exogenous ligand. Treatment with idebenone, CoQ_2_ or CoQ_10_ induces similar patterns of GFP expression in cells of the epidermis, blood, CNS and posterior spinal cord. Decylubiquinone, which carries a methyl group instead of the hydroxyl group, does not activate the receptor. Benzoquinone, CoQ_0_, CoQ_1_, CoQ_4_, CoQ_6_, CoQ_8_ and CoQ_9_ showed no reporter activation. Pure CoQ_10_ (Sigma), which has weak water solubility, shows strong receptor activation when mixed together with PEG. Overlay pictures of bright-field and GFP (85% transparent) of 2 dpf embryos are shown. Views are lateral with anterior to the left (mean *n*=20, replicated three times). (C) Idebenone and CoQ_10_ are partial dual agonists of PPARα and PPARγ. Embryos of the PPARδ fish line show no increased GFP expression in the presence of CoQ_10_ or idebenone. Overlaid bright-field and GFP images (85% transparent) of 2 dpf embryos are shown. Views are lateral with anterior to the left (mean *n*=10, replicated three times). (D) HEK293 cells were co-transfected with the GAL4-LBD fusion proteins of hPPARα, hPPARδ and hPPARγ (as indicated) together with a UAS-luciferase reporter. Treatment of the cells with the full agonists GW7647 (hPPARα), CAY10592 (hPPARδ/β) or rosiglitazone (hPPARγ) resulted in EC_50_ concentrations of 8 nM, 12.8 nM and 48 nM, respectively. Treatment with idebenone resulted in an EC_50_ of 2.5 µM for PPARα and 3.8 µM for PPARγ. Notably, maximal activation levels were far lower than elicited by the full agonists. Luciferase output was normalized to β-galactosidase to control for transfection efficiency and expressed as normalized luciferase output. Plasmids used were: pCMX, pcDNA3-GAL4-hPPARα, pcDNA3-GAL4-hPPARδ, pcDNA3-GAL4-hPPARγ, UAS-luc, pGEM, pCMX–β-galactosidase. Data represent mean±s.d., *n*=3 with at least three repeats.

Also identified was a novel PPARγ modulator, idebenone, which was first developed as a CoQ_10_ analog for potential use in Alzheimer's disease (Senin et al., 1992). In our assay, idebenone yielded maximal partial agonist activity when provided at a concentration of 3 µM (Fig. 2B). GFP expression was noticeably weaker and less widespread than the response to the full agonist rosiglitazone, indicating partial agonist activity with a more spatially restricted subset of responsive tissues (tail bud, forebrain and hindbrain for idebenone versus tail bud, brain, spinal cord and heart for TZDs; Figs 2C, 3B and Fig. S1). The PPARγ antagonist GW9662 was able to block this activation, consistent with direct competition within the LBD pocket (Fig. 2C). GW9662 did not block reporter gene expression elicited by the RXR agonist retinoic acid (Fig. 2B).

As described earlier, well-known side effects of TZDs include weight gain due to systemic increases in adipocyte numbers and lipid storage levels. Treating 3T3-L1 cells with rosiglitazone in culture has been previously used to model this effect (Chawla et al., 1994; Kliewer et al., 1995; Lehmann et al., 1997). As expected, rosiglitazone treatment resulted in the differentiation of 3T3-L1 pre-adipocytes to adipocytes, as indicated by the appearance of large Oil-Red-O-stained lipid vesicles (Fig. 2C and D). In contrast, idebenone treatment caused no significant increase in lipid content or vesicle size. Thus, as previously observed with other PPARγ partial agonists (Choi et al., 2011), idebenone does not stimulate adipocyte differentiation.

### Idebenone and other benzoquinones act as dual PPARα/γ partial agonists

Idebenone and CoQ_10_ share a benzoquinone (2,3-dimethoxy-5-methyl-*p*-benzoquinone) core with variable numbers of isoprenoid subunits in their side chains, as indicated by the subscript number (Fig. 3A). Initial treatments of the PPARγ LT zebrafish with pure CoQ_10_ resulted in only limited levels of activation in skin and gills. This weak response, as compared to idebenone, could be due to the well-known insolubility of CoQ_10_. Indeed, LipoQ, a commercially available, water-soluble version of CoQ_10_, induced GFP expression with spatial and quantitative responses comparable to idebenone (Fig. 3B). Since LipoQ also contains triglycerides, we also tested pure CoQ_10_ solubilized with polyethylene glycol 400 (PEG400), with similar results to those induced by LipoQ (Fig. 3B). We then tested other ubiquinones. Although the prototypical 1,4-benzoquinone did not induce a detectable GFP response, CoQ_0_, CoQ_1_, CoQ_4_, CoQ_6_, CoQ_8_ and CoQ_9_ all showed modest agonist activity, indicating that the 2,3-dimethoxy-5-methyl-*p*-benzoquinone moiety is necessary for PPAR activation. Interestingly, CoQ_2_ showed activity comparable to idebenone and water-soluble CoQ_10_ (Fig. 3B).

Next, we tested idebenone and CoQ_10_ on the PPARα and PPARδ receptors to test for specificity. While the PPARδ LT line showed no response to idebenone or CoQ_10_, the PPARα line showed relatively robust responses to both (Fig. 3C). We then compared our *in vivo* results with classical human cell-based assays to see whether the responses were similar. HEK 293 cells transfected with hPPAR expression vectors and corresponding luciferase reporters all showed responses above those of the reporter alone (Fig. 3D). As seen with our fish, idebenone and CoQ_10_ both elicited partial agonist transcriptional responses in PPARα- and PPARγ-transfected cells with similar EC_50_s to those observed in the zebrafish (Fig. 3D). Once again, PPARδ showed no response to idebenone or CoQ_10_ when tested in cultured cells.

### Idebenone and CoQ_10_ bind directly to PPARα and PPARγ LBDs

**Fig. 4.**
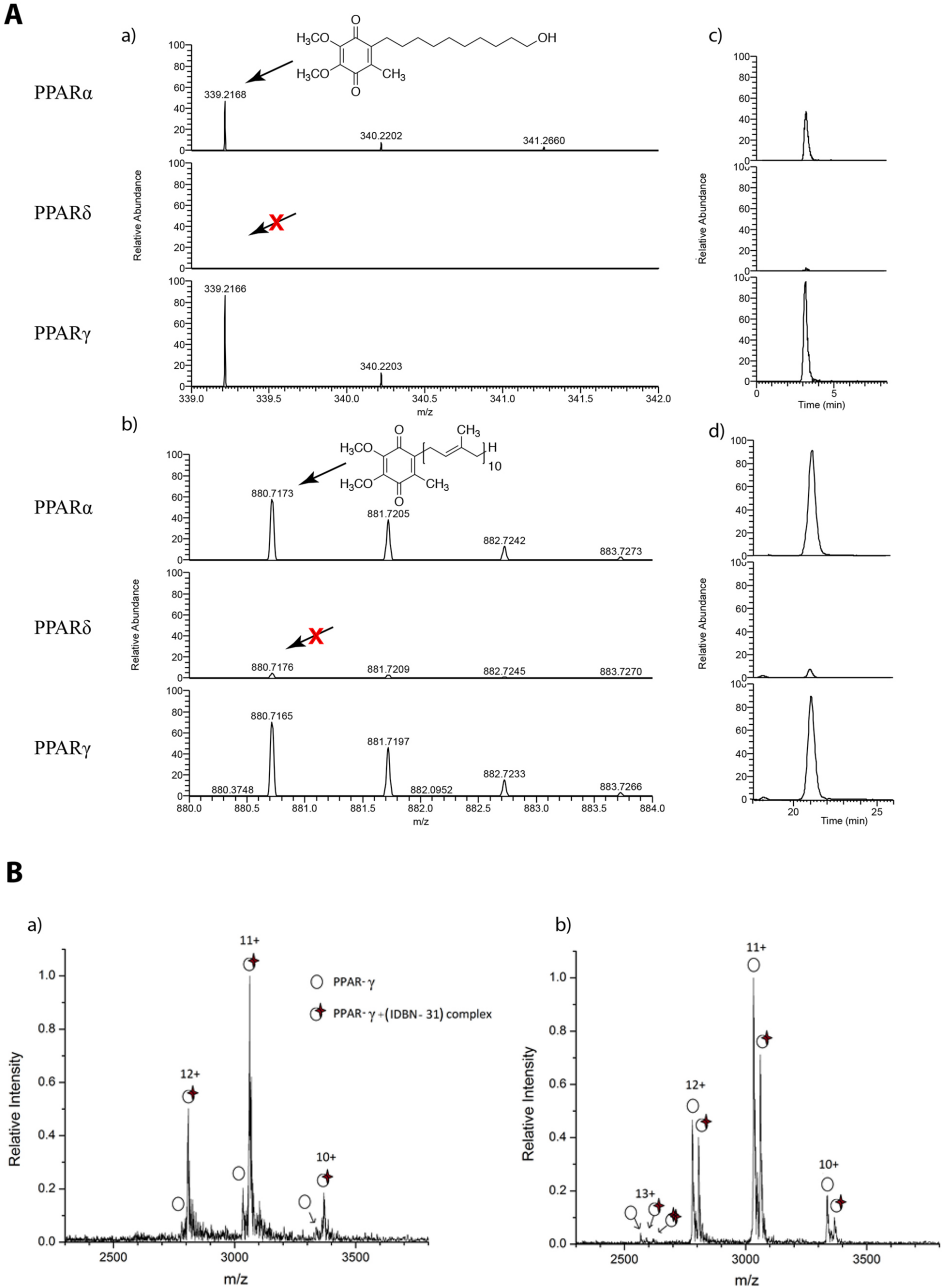
**Mass spectrometry of bound ligands.** (A) Liquid chromatography/ESI mass spectra under positive ionization mode. (a) Positive ESI-MS [M+H]^+^ of idebenone (IDBN) extracted from 2.5 mM treated PPAR LBDs. (b) Positive ESI-MS [M+NH_4_]^+^ of extract CoQ_10_ from 2.5 mM treatment PPARs LBDs. (c) HPLC chromatogram of idebenone mass extracted from a. (d) HPLC chromatogram of CoQ_10_ extracted from b. (B) NanoESI mass spectra of PPARγ-IDBN solution with desolvation capillary exit held at (a) 220 V (non-denaturing) and (b) 340 V (in-source CID) denaturing conditions. Panel a shows ionized masses corresponding predominantly to the bound PPARγ-IDBN complex, while panel b shows mass peaks corresponding primarily to unbound PPARγ.

To test whether idebenone and CoQ_10_ bind directly to PPAR LBDs, His-tagged PPARα, δ and γ LBDs were expressed in bacteria and incubated with 10 µM idebenone or CoQ_10_, followed by Ni-NTA affinity purification. After extensive washes, specifically-bound small molecules were extracted with organic solvent and analyzed by mass spectrometry (MS). These co-purifications confirmed strong binding of idebenone and CoQ_10_ to both the PPARα and PPARγ LBDs, but not to PPARδ (Fig. 4A). As an alternative test, non-denaturing mass spectra of the PPARγ LBD-idebenone complex was performed (Fig. 4B). Two masses were detected, with the smaller mass (marked with white circles) corresponding to the expected mass of the unbound LBD (calculated mass 33,349 Da; measured mass 33,347 Da), and the larger mass corresponding to the LBD mass plus an adduct of 307 Da. The latter corresponds to the mass of idebenone minus a 31 Da CH_2_O side group lost during the gas-phase activation process. The predominance of the ligand-bound complex indicates a stoichiometry of about 1:1. Following harsher collision-induced dissociation conditions, the adduct mass could be removed, giving rise to increased amounts of the unbound PPARγ LBD mass (Fig. 4B). Control nano-electrospray ionization (nanoESI) MS spectra obtained from LBD incubated in the absence of idebenone show no presence of a complex (Fig. S2).

### Idebenone regulates lipid metabolism in liver

**Fig. 5.**
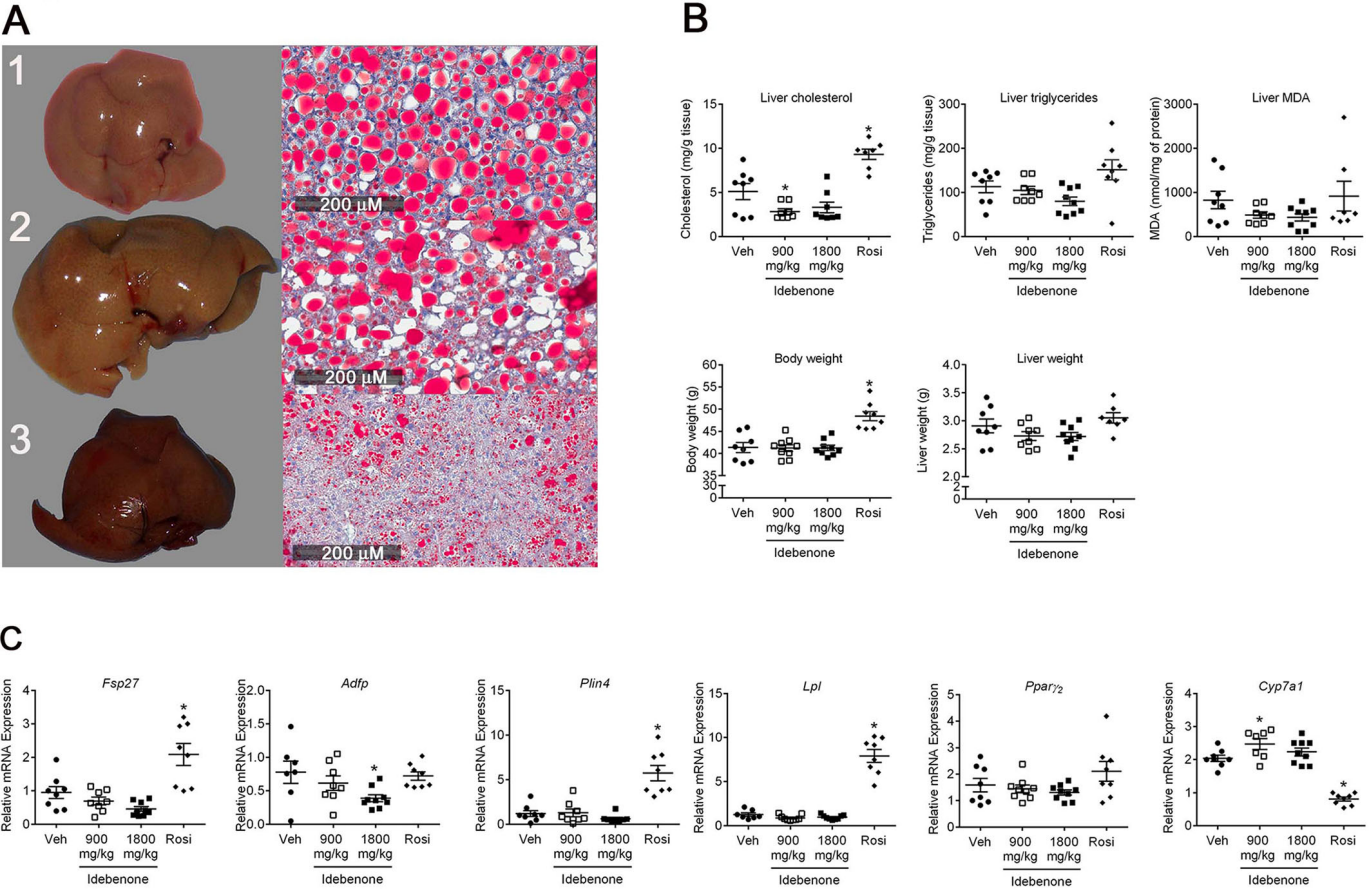
**Liver properties of treated *db/db* mice.** (A) *db/db* mice were treated for 21 days with either vehicle, 10 mg/kg rosiglitazone or 1800 mg/kg idebenone. Representative photomicrographs of livers and Oil-Red-O (ORO) staining of neutral lipids are shown for the three *db/db* mouse treatment groups: (1) vehicle; (2) rosiglitazone and (3) 1800 mg/kg idebenone. Lipid content was higher in vehicle- and rosiglitazone-treated *db/db* mice. (B) Total lipid analysis (triglycerides and cholesterol) from *db/db* livers and relative levels of reactive oxygen species detected using thiobarbituric-acid-reactive substances (TBARS/liver MDA). Body and liver weights were obtained at the end of the study. Data represent mean±s.d., *n*=7-9. *P<0.05 vs Veh; ANOVA followed by Holm-Sidak's test. (C) Relative mRNA expression levels of the PPAR target genes, *Fsp27*, *Adfp*, *Plin4*, *Lpl*, *Pparg2* and *Cyp7a1* as determined by qPCR. Data represent mean±s.d., *n*=7-9. *P<0.05 vs Veh; ANOVA followed by Holm-Sidak's test.

The partial agonist activity of idebenone on both PPARα and PPARγ, combined with its excellent safety profile in humans (Kutz et al., 2009), suggested a potential role in the treatment of metabolic syndrome diseases. Although much of orally consumed idebenone is broken down by first-pass metabolism in the liver of mice and humans (Kutz et al., 2009), we reasoned that, while sequestered in the liver and prior to being metabolized, it may still be effective in the treatment of fatty-liver-related diseases such as nonalcoholic fatty liver disease (NAFLD) and steatosis. To test this possibility, we examined the livers of leptin-deficient (*db/db*) mice fed on a normal diet supplemented with either vehicle (DMSO), rosiglitazone (10 mg/kg body weight) or idebenone (900 mg/kg or 1800 mg/kg). Although these doses may seem relatively high, they are only two to three times higher [after allometric scaling (Reagan-Shaw et al., 2008)] than doses used to treat patients with Friedrich's ataxia. Fig. 5A shows a typical fatty liver isolated from a vehicle-treated *db/db* mouse, with characteristic yellow appearance. Addition of rosiglitazone to the food over a 3-week period caused a further gain in liver weight, with even greater enlargement and yellowing (Fig. 5A,B, Table S2). In contrast, treatment with idebenone resulted in a reduction of liver weight and a healthy red color (Fig. 5A).

Oil-Red-O staining of sections from vehicle- and rosiglitazone-treated *db/db* mouse livers shows that lipid vesicle size is extremely large and lipid-dense. The idebenone-treated samples, on the other hand, showed dispersion of the enlarged vesicles into much smaller, largely empty vesicles, resulting in hepatocyte morphologies that more closely resemble those of wild-type liver hepatocytes (Fig. 5A). Similar results were observed with Toluidine-Blue-stained histological sections analyzed by bright-field microscopy (Fig. S3). Analysis of mitochondria morphologies via electron microscopy (Fig. S3) also indicated significant improvements after idebenone treatment. Christae morphology was relatively uniform as opposed to the irregular densities and membrane lesions observed with vehicle- and rosiglitazone-treated liver mitochondria.

Liver histological assays also showed decreased levels of cholesterol and triglycerides in comparison to those of rosiglitazone-treated mice (Fig. 5B, Table S2A-D). Corresponding reductions in liver and body weight were also reduced for the idebenone-treated mice. Oxidative stress is another measure of pathogenesis involved in liver steatosis. To test for this, and possible effects by rosiglitazone and idebenone, we assayed the levels of thiobarbituric-acid-reactive substances (TBARS) in liver homogenates. The results show high levels of lipid peroxides in all samples, with somewhat lower levels in the idebenone-treated *db/db* mice (liver MDA, Fig. 5B).

### Idebenone regulates key PPAR target genes in liver involved in lipid metabolism

To understand the functional and regulatory mechanisms of idebenone in treated *db/db* mice, we analyzed gene expression in the vehicle- and drug-treated liver samples obtained at the completion of the treatment program. Although no longer representing the acute phase of drug responses, significant changes in lipid-regulating target genes were observed. Examples include *Fsp27/Cidec*, *Adfp*, *Plin4*, *Pparg2*, *Lpl* and *Cyp7a1* (Fig. 5C). All of the genes downregulated by idebenone are lipogenic, while the upregulated gene *Cyp7a1* may also partially explain the lower levels of cholesterol, as *Cyp7a1* is the rate-limiting enzyme in the conversion of cholesterol into bile acids.

## DISCUSSION

This study marks the first *in vivo* screen for selective PPAR small-molecule regulators. Our identification of CoQ_10_ and its more soluble analog idebenone as partial agonists for both PPARα and PPARγ provides new insight into PPAR molecular and genetic functions, as well as promising new reagents for treating and preventing metabolic disorders. CoQ_10_ and idebenone belong to the quinoid-ring-containing benzoquinones (O'Brien, 1991). As partial agonists of PPARα and PPARγ, CoQ_10_, idebenone and other related benzoquinones have the potential to treat a variety of metabolic disorders.

### Mechanisms of idebenone action in fatty livers

Using idebenone to treat diabetic mice, we found it to be very effective in lowering hepatic lipid levels. Liver steatosis is characterized by excessive accumulation of neutral lipids, mainly triglycerides, into intracytoplasmic macrovesicles whose formation can be induced by various factors, including TZDs (Donato and Gomez-Lechon, 2012). Histological analyses of *db/db* murine livers showed significantly more abundant and larger lipid-filled vesicles in both vehicle- and rosiglitazone-treated mice. In contrast, the idebenone-treated livers showed breakdown of the enlarged vesicles, resulting in hepatocyte morphologies that more closely resemble those of wild-type livers.

Previous studies have suggested that antioxidants may have a general ability to reduce liver fat content. While this may be possible, our results with CoQ_10_ and idebenone, both of which are strong antioxidants, suggest that they act primarily via the regulation of PPAR target genes. These include the important lipogenic regulators *Fsp27*, *Adfp*, *Plin4*, *Ppar*γ*2* and *Cyp7a1*. These changes correlate well with the observed reduction in hepatic triglyceride and cholesterol levels. In particular, it has been shown recently that *Fsp27*, on its own, not only promotes lipid droplet stability but also inhibits lipolysis (Akinrotimi et al., 2017; Langhi and Baldan, 2015). Even more striking is that increased expression of *Fsp27* is sufficient to promote hepatic steatosis, with liver morphologies highly similar to those found here in vehicle- and rosiglitazone-treated *db/db* mice (Guillen et al., 2009). Given that these downregulated genes are targets of PPARγ activation, we presume that their downregulation is not brought about by activation of liver PPARγ, which typically promotes adipogenesis (Davies et al., 1999; Okumura, 2011). This interpretation is also consistent with the observation that PPARγ knockout animals are resistant to fatty liver (Matsusue et al., 2003), that idebenone treatment reduced levels of *Ppar*γ*2* expression, and treatment with the strong PPARγ agonist rosiglitazone led to higher levels of fat in treated *db/db* mice. Taken together with the fact that normal PPARγ expression in livers is much lower than PPARα (Wang, 2010), we suggest that the effects of idebenone are mediated primarily via PPARα activation.

### CoQ_10_ as an endogenous PPAR ligand

Given the roles that PPARs play in controlling mitochondria and peroxisome abundance and activities (Corona et al., 2014; Miglio et al., 2009), and the major roles played by CoQ_10_ in both organelles, our discovery of a direct interaction with both the PPARα and PPARγ LBDs suggests a logical role as a status indicator for one or both organelles. PPARα has also been shown to regulate CoQ_10_ biosynthesis (Turunen et al., 2000), suggesting another role for feedback regulation. The connection between PPARs and the mitochondria-located uncoupling proteins (UCPs) is also highly intriguing. All three PPARs are transcriptional activators of UCP genes. Like the PPARs, UCPs are also activated by fatty acids and promote their metabolism via β-oxidation (Villarroya et al., 2007). Notably, UCPs also require CoQ_10_ as an obligatory, non-covalently bound cofactor. In brown fat, PPARγ activates expression of UCP1, resulting in the generation of heat (Kelly et al., 1998). In hepatocytes, PPARα induces UCP2 expression following high-fat meals or upon treatment with PPARα agonists or polyunsaturated fatty acids (Villarroya et al., 2007). Thus, in liver, idebenone and CoQ_10_ may be coordinating lipolysis and β-oxidation of fatty acids via simultaneous regulation of PPARα and its UCP target gene proteins.

Interestingly, CoQ_10_ has features in common with another nuclear receptor ligand that we recently identified, the NR1D ligand heme (Pardee et al., 2009; Reinking et al., 2005). Like heme, CoQ_10_ provides the potential to monitor and control cellular metabolism, redox state and hypoxia. In the case of PPARs, we found that only the reduced form of CoQ_10_ can bind and activate the receptors.

One of the questions raised by the potential role of CoQ_10 _as a PPAR ligand is how it would come into contact with PPAR proteins. As with other NR ligands, CoQ_10_ may associate with transporter proteins that carry it to the nucleus. Two proteins that have been shown to bind CoQ_10_ are Coq10p (Cui and Kawamukai, 2009) and saposin B (Jin et al., 2008). Alternatively, PPARs may shuttle to cytoplasmic compartments where CoQ_10_ is more abundant. Notably, previous studies have found PPARγ, PPARδ, RXRs and the PPAR coactivator protein PGC-1α bound to the D-loop promoter within mitochondria, and to be associated with increased mitochondrial gene expression (Dominy and Puigserver, 2013; Puigserver, 2005). An ability of PPARs to regulate the expression of both nuclear and mitochondrial genomes would provide an obvious means of coordinating the two programs. How these programs are coordinated is an important problem, as loss of this coordination has been linked to many neuronal, muscular and aging-related disorders (Scarpulla, 2008).

Given that CoQ isoforms with shorter isoprenyl chains also have partial agonist activity, these isoforms could also serve as natural PPAR ligands. These have been documented at low concentrations in various tissues, perhaps representing breakdown or precursor forms of CoQ_10_. It is also possible that shorter versions from other organisms, such as bacterial CoQ_8_, might serve as functional ligands. This could occur in the gut, for example, where bacteria have been shown to modulate inflammatory responses by promoting the transport of PPARγ out of the nucleus (Annese et al., 2012).

Another question is how the long CoQ_10_ isoprenyl tail might fit within PPAR LBD pockets. This could readily be achieved if the tail folds via hydrophobic interactions. Indeed, folding predictions suggest a globular structure with a total volume of 352 Å^3^ (Di Bernardo et al., 1998), which is well within the size range for PPAR ligands. The PPARγ LBD pocket, for example, is∼1300 Å^3^ in the apo form (Nolte et al., 1998).

### Other potential therapeutic roles for benzoquinones

PPARs are involved in a number of metabolic, inflammatory and bone disorders in addition to T2D and fatty liver disorders. Idebenone has also been shown to reduce the growth and viability of a number of cancer cell lines (https://dtp.cancer.gov/discovery_development/nci-60/). Presumably, CoQ_10_ and idebenone could be used to treat some of these disorders. However, CoQ_10_ lacks sufficient solubility to be used effectively either orally or topically for any of these indications. Idebenone use is also compromised by its high first-pass metabolism rates in the liver. These limitations might be countered via the use of alternative methods of administration, improved solubility or through chemical variations that prevent hepatic metabolism.

## MATERIALS AND METHODS

### Transgenic fish line generation and maintenance

Zebrafish (AB/Tübingen) were maintained at 28.5°C on a 14/10 h light/dark cycle and staged according to hours post-fertilization (hpf) or dpf. The generation of new pLT PPARα (aa 179-468), PPARδ (aa 142-441) and PPARγ (aa 189-477) plasmids and transgenic lines were created as previously described (Tiefenbach et al., 2010).

F0 fish were crossed with wild-type (WT) fish to identify germline transformed animals, as determined by agonist treatments and GFP expression. F1 progeny showing strong and consistent GFP responses were selected for F2 homozygote production. To avoid reporter GFP silencing of stable transgenic LT lines, homozygous fish showing strong and consistent GFP responses were selected for further propagation. Homozygous fish were crossed to *casper* fish, which lack melanocytes and iridophores in both embryogenesis and adulthood (White et al., 2008).

### Compound screening

PPARγ zebrafish screens were performed under optimized conditions in 96-well format. 1 dpf PPARγ heterozygous embryos were heat induced (28→37°C) for 30 min, dechorionated and then arrayed in 96-well plates (5 per well). Screens were performed in duplicate. Embryo water (0.075 g/l NaHCO_3_, 0.018 g/l sea salt, 0.0084 g/l CaSO_4_.2H_2_O) was removed and 200 µl of fresh embryo water/well, including dissolved small molecules or solvent, was added. The Enzo FDA-approved drug library (BML-2841-0100; 640 compounds) was screened at 1 µM concentration. Embryos were incubated at 28°C for 14 h, anesthetized with Tricaine (Sigma, cat. # A-5040) and then analyzed for GFP fluorescence using an ImageXpress Velos Laser Scanning Cytometer. GFP expression signals in embryos were compared to vehicle-treated embryos. An increase in fluorescent signal indicated an agonistic activity of the test compound. Positive hits were re-screened at 3 and 10 µM under the same conditions described above. Lethality and toxicity levels were also recorded.

### Murine 3T3-L1 adipocyte differentiation assay

Murine 3T3-L1 cells were obtained from the American Type Culture Collection (ATCC) and maintained in fibroblast state in DMEM (Gibco: 11995-065), 10% fetal bovine serum (FBS; Gibco: 26140-079) and Pen-Strep solution (Gibco: 15140-122). Cells were grown in 75 cm^2^ flasks and sub-cultured just prior to confluence. Culture medium was then removed and cells washed twice with PBS. For cell differentiation, 2 day post-confluent monolayers were incubated for 48 h in DMEM containing 10% FBS, 100 µg/ml IBMX (from a 500× stock in 0.15 M KOH, prepared fresh), 390 ng/ml dexamethasone (from a 1000× stock in EtOH) and 5 µg/ml insulin (from a 1000× stock in 0.1 M HCl) in the presence or absence of 1 µM rosiglitazone (RGZ) or 25 µM idebenone (IDBN). After 2 days, media was replaced with DMEM+10% FBS and 5 µg/ml insulin and 1 µM rosiglitazone or 25 µM idebenone only, and cells were maintained for another 2 days. Media was then replaced by DMEM+10% FBS and 1 µM rosiglitazone or 25 µM idebenone for 5-6 days until differentiation was complete. After differentiation, media was removed and cells were washed twice with PBS and then fixed in 10% neutral buffered formalin (Sigma) for 1 h at room temperature. Cells were stained with 0.6% (w/v) Oil-Red-O solution (60% isopropanol, 40% water) for 10 min at room temperature, followed by five washes with ddH_2_O to remove unbound dye, and images were taken with a Leica M205 FA microscope.

### Cell culture reporter assays

Human embryonic kidney 293 (HEK293) cells were obtained from ATCC and maintained in DMEM, 10% FBS and Pen/Strep. Transfection was carried out using calcium phosphate with indicated expression constructs and UAS-luciferase reporter as previously described (Makishima et al., 1999). Treatment with ligands was carried out 1 day after transfection. After 48 h, cells were lysed. Luciferase values were normalized for transfection efficiency using β-galactosidase. Plasmids used were: pCMX, pcDNA3-GAL4-hPPARα, pcDNA3-GAL4-hPPARδ/β, pcDNA3-GAL4-hPPARγ (aa 188-477), UAS-luc, pGEM and pCMX–β-galactosidase.

### Chemicals

Suppliers and chemicals used were: Alexis Biochemicals: pioglitazone (ALX-270-367); Cayman Chemicals: rosiglitazone (#71740), GW7647 (#10008613) and CAY10592 (#10012536); Sigma-Aldrich: idebenone (#I5659), CoQ_0_ (#D9150), CoQ_1_ (#C7956), CoQ_2_ (#C8081), CoQ_4_ (#C2470), CoQ_9_ (#27597), 1,4-benzoquinone (#51386), dimethyl sulfoxide (#D8418); Bioshop: acetone (#ACE888.500); Inno-Vite Inc.: CoQ_10_ Li-Q-Sorb (NPN80007078), polyethylene glycol BioUltra, 400 (synonym: PEG; #91893), Avanti CoQ_8_ (#900151) and CoQ_6_ (#900150).

### Protein purification

Proteins expressed in *E. coli* were purified using Ni-NTA affinity chromatography as described previously (Pardee et al., 2009).

### Mass spectrometry analysis of extracted small molecules

Idebenone and CoQ_10_ stock solutions were prepared to a concentration of 50 mM using DMSO. The PPAR LBDs (∼100 μM) from the purifications described above were incubated with 2.5 mM ligand for 12 h at 25°C. LBD-ligand complexes were then loaded onto a PD-10 desalting column containing 8.3 ml of Sephadex G-25 medium (GE Healthcare) and eluted with 5 mM Tris-HCl pH 7.5. Eluates were extracted with 2:1 chloroform/methanol, and the chloroform phase collected and dried under a stream of nitrogen gas. Dried residues were reconstituted in isopropanol for LC-MS. Idebenone was analyzed as H^+^ adducts, and CoQ10 as NH_4_^+^ adducts using positive ion ESI/MS on a Thermo Fisher Q Exactive HF Hybrid Quadrupole-Orbitrap Mass Spectrometer controlled by Xcalibur software. Samples were loaded into the UPLC system in isopropanol and the reverse phase column (Polaris 3 NH_2_ 100×2.0 mm, Agilent Technologies), and eluted with a gradient of 100% buffer A (methanol: acetonitrile: H_2_O=6:2:2 containing 1 mM ammonium acetate), 0% buffer B, at time zero, with a flow rate of 0.2 ml/min, kept isocratic for 2 min, increased to 100% buffer B (100% ethanol containing 1 mM ammonium acetate) by 17 min, kept stable for 7 min, then returned to 100% buffer A over the next 5 min.

### Mass spectrometry analysis of native LBD-ligand complexes

Mass spectra were acquired on a 7.0T Fourier Transform Ion Cyclotron Resonance (FT-ICR) mass spectrometer (Bruker Apex Qe; Bruker Daltonics, Billerica, MA, USA) equipped with a nanoESI source. For nanoESI-MS compatibility, PPARγ-IDBN samples were purified using Superdex 200 gel filtration columns followed by four cycles of desalting with 10-kDa cut-off centrifugal filters (Amicon Ultra-15) into pH 6.2, 20 mM ammonium acetate until a concentration of ∼1 mg/ml was obtained, as verified by the Bradford assay. Samples were then diluted to ∼10 µM in the same solvent. The ESI solution was loaded into nano-ESI emitters, which were formed by pulling borosilicate glass capillaries to a fine (∼3 µm) orifice. A grounded platinum wire was inserted into the loaded capillary emitter while the entrance of MS was held at –0.9-1.0 kV to generate positive-mode ESI. MS source and instrument conditions were optimized to preserve the complex. Key conditions included maintaining the MS desolvation capillary at a low temperature (100°C), use of increased pressure (4.3 mbar) in the first vacuum stage of mass spectrometer (accomplished by constricting the flow to the vacuum pump that evacuates this region) and argon gas flow (0.1 l/s) into the ion accumulation cell to collisionally cool the complex. In some experiments, in-source collision-induced dissociation (CID) was performed by increasing the desolvation capillary exit voltage from 220 V to 340 V, while keeping all other potentials constant. Mass spectra shown correspond to the sum of 100 mass spectra, each with 64,000 data points at a sampling frequency of 723 kHz. Deconvoluted average masses were calculated using DataAnalysis version 3.3 (Bruker Daltonics).

### Mouse studies

Male C57BL/6 *db/db* mice [B6.BKS(D)-Lepr db/J] were ordered from Jackson Laboratories (#000697) at 7 weeks of age. Mice were housed individually with free access to food (2016 Teklad Global 16% Protein Rodent Diet) and water on a 14-h light/dark cycle (6 a.m. to 8 p.m. light). 10% sucrose and 100 µl Tween-80 was added to 300 g food that was supplemented with either vehicle (DMSO), 10 mg/kg body weight rosiglitazone, 900 mg/kg idebenone or 1800 mg/kg idebenone. Food was exchanged daily. Mice were randomized by body weight into four treatment groups (*n*=8). Dosing occurred for 21 days. Mice were euthanized by exsanguination under anesthesia with isoflurane and livers were fixed in neutral buffered formalin for 24 h. The next day, samples were transferred to 10% sucrose (for another 24 h) and then 30% sucrose. Oil-O-Red staining was performed through the Pathology Research Program at the University Health Network in Toronto.

### TBARS assay

Thiobarbituric acid reactive substances (TBARS) were measured as a surrogate marker of oxidative stress. 100 mg of *db/db* livers were homogenized in RIPA buffer, and protein concentration was quantified using a BCA protein assay kit (Cell Signaling, #7780) followed by measurement using a TBARS assay kit according to the manufacturer's instructions (Cayman Chemicals, #10009055).

### Enzymatic tissue cholesterol and triglyceride assays

Liver lipids were extracted using the Folch method as previously described (Patel et al., 2011). Briefly, liver tissue (∼100 mg) was homogenized in chloroform/methanol (2:1 v/v), washed once in 50 mM NaCl and twice in 0.36 M CaCl_2_/methanol. The organic phase was separated and brought up to 5 ml with chloroform. Dried aliquots of standards and samples were re-dissolved in 10 µl of 1:1 cholorform:Triton X-100 and evaporated overnight. Samples were assayed for cholesterol and triglycerides using commercial colorimetric assays (Thermo).

### Microscopy

Tissues were fixed in 2.5% glutaraldehyde in a 0.1 M cacodylate buffer and post-fixed in OsO_4_ (2%) in a 0.1 M cacodylate buffer. After washing in 0.1 M cacodylate buffer, the cells were dehydrated through an ethanol series. The material was then penetrated by an increasing series of Spurr resin diluted in acetone. Finally, the tissues were embedded in Spurr resin. Both the thick and ultrathin sections were sectioned with an Ultracut Leica UC6 ultramicrotome (Leica Microsystems, Germany) using a diamond knife. Thick sections (0.5 μm) were placed on the slides, stained with Toluidine Blue, and imaged with a Leica DMRA2 fluorescence microscope using a Q-Imaging Retiga EX camera and Openlab 3.1.7 software. Ultrathin sections (80 nm) were placed onto copper grids coated with Formvar film, stained with uranyl acetate and lead citrate solutions, and examined with a Zeiss Libra 120 transmission electron microscope (Karl Zeiss Group, Germany).

### RNA isolation, cDNA synthesis and real-time qPCR analysis

Total RNA was extracted from 100 mg of *db/db* livers using RNA STAT-60 (Tel-Test, Inc.), treated with DNase I (RNase-free, Roche) and reverse transcribed into cDNA with random hexamers using the High Capacity Reverse transcription system (Applied Biosystems, ABI). Primer sequences are listed in Table 1. Real-time qPCR reactions were performed on an ABI 7900 in a 384-well plate containing 12.5 ng cDNA, 150 nM of each primer, and 5 μl 2× SYBR Green PCR Master Mix (ABI) in a 10 μl total volume. Relative mRNA levels were calculated using the comparative Ct method normalized to cyclophilin mRNA.

**Table 1.**
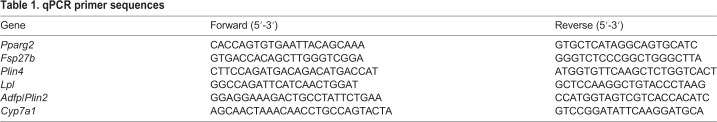
**qPCR primer sequences**

## Supplementary Material

Supplementary information

First Person interview
