# Supplementary material for: Idebenone and coenzyme Q10 are novel PPARα/γ ligands, with potential for treatment of fatty liver diseases
Source: Dis Model Mech. 2018 Aug 31;11(9):dmm034801. doi: 10.1242/dmm.034801 (PMC6177011; doi:10.1242/dmm.034801)
Supplement: Supplementary information [file dmm-11-034801-s1.pdf]

FIRST PERSON

First person – Jens Tiefenbach

First Person is a series of interviews with the first authors of a selection of papers published in Disease Models & Mechanisms, helping early-career researchers promote themselves alongside their papers. Jens Tiefenbach is first author on 'Idebenone and coenzyme Q_{10} are novel PPARα/γ ligands, with potential for treatment of fatty liver diseases', published in DMM. Jens is a Research Associate in the lab of Henry Krause at University of Toronto, ON, Canada, investigating the interplay between transcription, protein regulation and small molecule modulations in the context of human health.

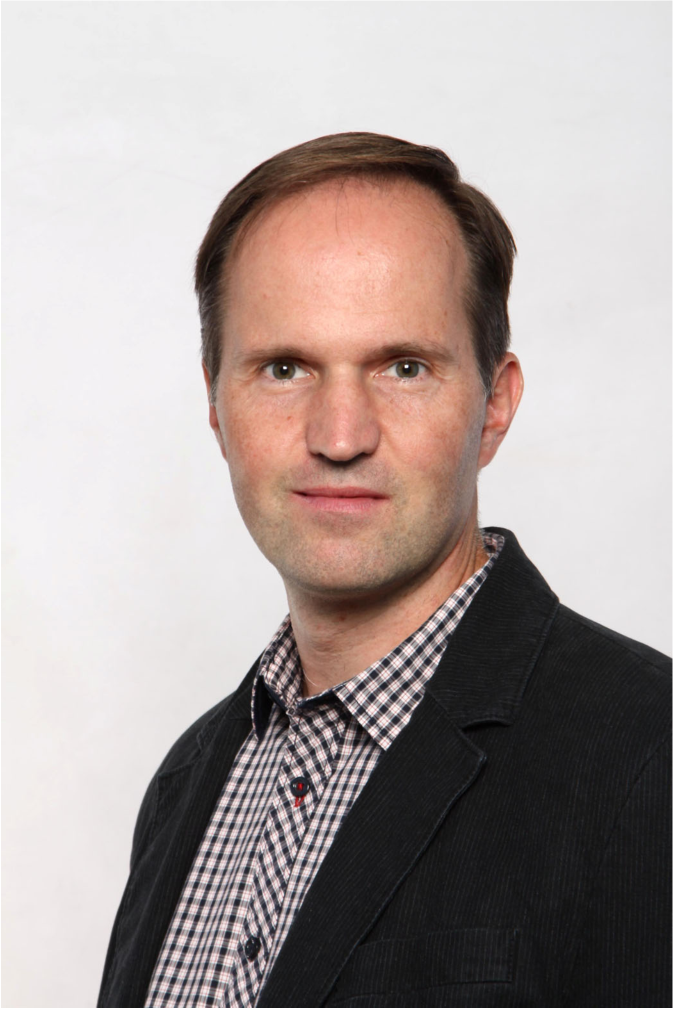

Jens Tiefenbach

How would you explain the main findings of your paper to non-scientific family and friends?

Nonalcoholic fatty liver disease (NAFLD) is a condition in which fat builds up in your liver, resulting in inflammation and liver cell damage. A healthy lifestyle and exercise is the best prescription to prevent this from happening, but NAFLD is on the rise worldwide. Currently, there are no drug therapies for this condition. Drugs that target a protein called peroxisome proliferator-activated receptor (PPAR) have potential to improve liver function and reduce fat build up, but the drugs currently available are associated with undesirable side effects in other tissues. We generated a zebrafish model that can identify new tissue-selective potential drugs to weakly target PPAR with fewer toxic effects. We found a drug, idebenone, with structural similarities to coenzyme Q_{10}, which has weak activity against two forms of the PPAR protein, PPARα and PPARγ. Testing idebenone in a mouse model of type 2 diabetes revealed the ability to reverse fatty liver development. This partial activity of idebenone against both PPARα and PPARγ, combined with its excellent safety profile in humans, demonstrates that it could be a good treatment option for nonalcoholic fatty liver disease (NAFLD).

> "*In vivo* [zebrafish] screens allow the identification of tissue-selective bioactive small molecules with good permeability, uptake, stability and delivery."

What are the potential implications of these results for your field of research?

In drug discovery, the key challenge is selecting the right assay and finding compounds that can reach, and function specifically, within the intended cells, considering drug absorption, distribution and metabolism. Commonly used assays in cells or *in silico* cannot assess these properties. We developed a whole-animal assay that allows identification of selective compounds and provides information on a drug's effectiveness, selectivity and toxicology, as well being highly cost-effective in a live vertebrate model. We validated our zebrafish ligand trap technology and tested our findings in a mouse model of type 2 diabetes. Our results will hopefully encourage other researchers to perform screens in whole-animal models and test their findings in rodent or clinical studies. We hope that this will identify drugs that have better chances of success in clinical trials.

What are the main advantages and drawbacks of the model system you have used as it relates to the disease you are investigating?

The zebrafish (*Danio rerio*) is an emerging vertebrate model for drug discovery that allows whole-animal drug screening. There are many advantages of the model. *In vivo* screens allow the identification of tissue-selective bioactive small molecules with good permeability, uptake, stability and delivery. Embryos develop *ex utero* and are optically clear, which allows the use of fluorescent reporters to pinpoint receptor activity in live animals. Embryogenesis is completed after 3 days of development and the cost to maintain zebrafish is a fraction of that of rodents or other mammalian organisms. The zebrafish model also has some disadvantages. Several mammalian organs are not present in the zebrafish, including breast tissue, lungs

Jens Tiefenbach's contact details: University of Toronto, 160 College St, Toronto, ON M5S 3E1, Canada.
E-mail: j.tiefenbach@indanio.com

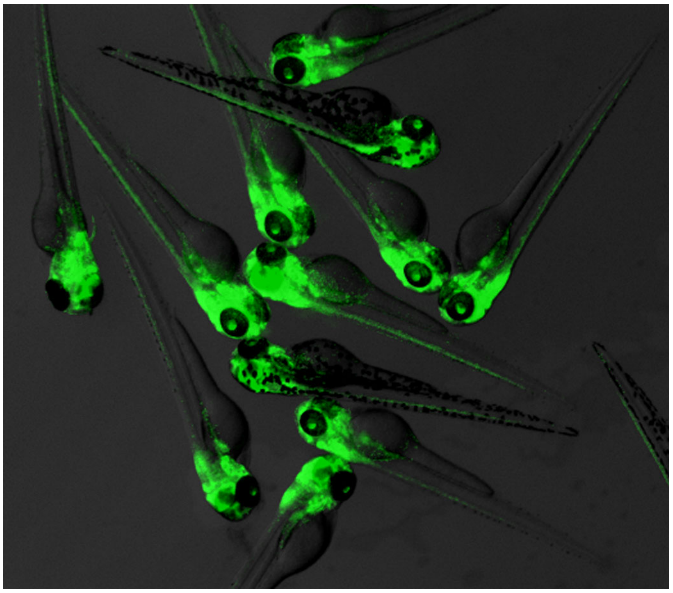

Nuclear receptor ligand trap zebrafish larvae. Expression of nuclear GFP is used to monitor receptor activity in a tissue-specific manner in live zebrafish.

and prostate. The biggest disadvantage for establishing transgenic zebrafish lines is that it takes a long time, as sexual maturation is reached in 3-4 months. For screening a fluorescent reporter fish line, embryos need to have uniform GFP expression. This can only be achieved with stable homozygous lines and the generation of these can take up to 2 years (5 generations).

What has surprised you the most while conducting your research?

I was most surprised by how powerful small molecules are in biology. Of course, this seems obvious, but when you study small molecules in a live transparent zebrafish embryo with fluorescent reporters you can monitor their influence on your target receptor almost in real time. Using a potent agonist in picomole concentrations, you might be under the impression there is nothing left of that compound after you did the serial dilution, but then you obtain a strong tissue-selective response. Compared to results from luciferase assays, which give you numbers, looking at the glowing embryo and monitoring the tissue-selective response is very impressive and can be surprising.

Describe what you think is the most significant challenge impacting your research at this time and how will this be addressed over the next 10 years?

The most significant challenge in drug development is to advance discovery in a way that leads to more FDA-approved drugs. Next to regulatory aspects of this timely process, I believe that selecting a drug candidate from a whole-animal model will help to improve success rates and reduce cost and time. In addition, access to good small molecule libraries is key. Purchasing drug libraries or analog molecules can be very expensive, which can delay research or result in termination of projects. Drug development would also benefit from a pooling of resources in, for example, an international drug resource center, and more open interactions between pharma labs and academic labs.

What changes do you think could improve the professional lives of early-career scientists?

I think early professionals would benefit from more funding and fellowship programs granted to them directly. The application process is good training for their later careers and it would allow them to develop their own innovative ideas and try new technologies in their projects. Universities should also train scientists for a life outside academia. Mentoring throughout their training is important and will help them to find the right path for their future.

> **"[…] when you study small molecules in a live transparent zebrafish embryo with fluorescent reporters you can monitor their influence on your target receptor almost in real time."**

What's next for you?

In addition to my interest in metabolic syndromes, I am interested in the relationship between health, lifestyle, sleep and circadian rhythm in the development of human diseases. Novel drug-discovery techniques targeting cellular circadian rhythm in a specific disease background are needed to improve the treatment options for many major diseases such as depression, autism and drug addiction, as well as certain types of cancer. Recently, I developed assays that relate, in part, to the discovery that the activity of a clock protein is surprisingly impaired under conditions of disrupted circadian rhythm when compared with its activity levels under normal conditions. This discovery was transferred into screening assays in our fish model. Initial screens identified drugs that work under either normal or disrupted rhythm. I now have completed my term at the University of Toronto and incorporated a life science company with a focus on chronobiology disorders. My aim is to discover selective drugs for clock protein-related diseases to help provide solutions for the treatment of autism, addiction and other neurological disorders.

Reference

Tiefenbach, J., Magomedova, L., Liu, J., Reunov, A. A., Tsai, R., Eappen, N. S., Jockusch, R. A., Nislow, C., Cummins, C. L. and Krause, H. M. (2018). Idebenone and coenzyme Q_{10} are novel PPARα/γ ligands, with potential for treatment of fatty liver diseases. *Dis. Model. Mech.* **11**: dmm0348010.
